# Transmembrane and coiled‐coil 2 associates with Alzheimer's disease pathology in the human brain

**DOI:** 10.1111/bpa.13290

**Published:** 2024-07-31

**Authors:** Paul C. R. Hopkins, Claire Troakes, Andrew King, Guy Tear

**Affiliations:** ^1^ Centre for Developmental Neurobiology King's College London London UK; ^2^ London Neurodegenerative Diseases Brain Bank Institute of Psychiatry, Psychology and Neuroscience, King's College London London UK

**Keywords:** Alzheimer's disease, amyloid protein precursor (APP), apolipoprotein E (apoE), transmembrane and coiled‐coil 2 (TMCC2)

## Abstract

Transmembrane and coiled‐coil 2 (TMCC2) is a human orthologue of the *Drosophila* gene *dementin*, mutant alleles of which cause neurodegeneration with features of Alzheimer's disease (AD). TMCC2 and Dementin further have an evolutionarily conserved interaction with the amyloid protein precursor (APP), a protein central to AD pathogenesis. To investigate if human TMCC2 might also participate in mechanisms of neurodegeneration, we examined TMCC2 expression in late onset AD human brain and age‐matched controls, familial AD cases bearing a mutation in APP Val717, and Down syndrome AD. Consistent with previous observations of complex formation between TMCC2 and APP in the rat brain, the dual immunocytochemistry of control human temporal cortex showed highly similar distributions of TMCC2 and APP. In late onset AD cases stratified by APOE genotype, TMCC2 immunoreactivity was associated with dense core senile plaques and adjacent neuronal dystrophies, but not with Aβ surrounding the core, diffuse Aβ plaques or tauopathy. In Down syndrome AD, we observed in addition TMCC2‐immunoreactive and methoxy‐X04‐positive pathological features that were morphologically distinct from those seen in the late onset and familial AD cases, suggesting enhanced pathological alteration of TMCC2 in Down syndrome AD. At the protein level, western blots of human brain extracts revealed that human brain‐derived TMCC2 exists as at least three isoforms, the relative abundance of which varied between the temporal gyrus and cerebellum and was influenced by APOE and/or dementia status. Our findings thus implicate human TMCC2 in AD via its interactions with APP, its association with dense core plaques, as well as its alteration in Down syndrome AD.

## Graphical abstract

Transmembrane and coiled‐coil 2 (TMCC2) is an apolipoprotein E‐binding protein with an evolutionarily conserved interaction with the amyloid protein precursor family, mutation of whose *Drosophila* orthologue causes neurodegeneration with features of Alzheimer's disease (AD). We investigated TMCC2 in late onset AD as well as early onset AD associated with disruptions to the amyloid protein precursor. We found that TMCC2 immunoreactivity is associated with the dense‐cored plaques of AD pathology and is additionally found in thread‐like pathological features in Down syndrome AD.

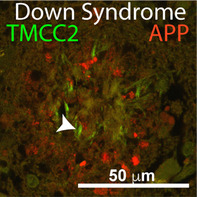

## 1 INTRODUCTION

Transmembrane and coiled‐coil 2 (TMCC2) forms an evolutionarily conserved complex with the amyloid protein precursor (APP) [1, 2], the source of the Aβ peptide whose accumulation as amyloid plaques in the brain defines Alzheimer's disease (AD). The mechanisms by which Aβ accumulation is associated with neurodegeneration in late onset AD remain debated. Aβ is produced by the intracellular endoproteolysis of APP and accompanied by the production of additional APP fragments, such as secreted APP and the C‐terminal 99‐amino acid fragment, which are also implicated in neuronal homeostasis and degeneration.

The *Drosophila* orthologue of TMCC2, called Dementin, protects against developmental and behavioural defects caused by ectopic expression of human APP in flies [2]. Conversely, mutated Dementin causes neurodegeneration with features of AD, that is, perturbed metabolism and proteolysis of the *Drosophila* APP homologue, the APP‐like protein, cytoskeletal and synaptic defects, and early death [2].

TMCC2 is expressed by mouse primary neurons and endogenous rat TMCC2 forms complexes with rat APP in brain extracts [1]. Human TMCC2 shows binding to apolipoprotein E (apoE), the ε4 isoform of which (apoE4) is the greatest risk factor for late onset AD after age [3, 4]. TMCC2 mediates an apoE‐dependent increase in Aβ secretion from cells expressing an autosomal dominant variant of APP, APP‐Swedish (K595N and M596L), as well as from cells expressing the C‐terminal fragment of APP that is generated in vivo following cleavage by BACE, with apoE4 having a greater effect than the most common apoE isoform, apoE3 [1].

Taken together, the above prior findings in vitro and in *Drosophila* suggested that TMCC2 would demonstrate an interaction with APOE genotype and/or an association with AD pathology. Here, we test this hypothesis and report the first investigation of TMCC2 expression in the human brain. We examined TMCC2 immunoreactivity in late onset AD cases stratified by APOE genotype and age‐matched non‐demented controls, as well as in early onset AD cases associated with mutations in APP or Down syndrome. We found that TMCC2 associates with characteristic pathologies of AD in both early and late onset AD, and shows variation in apparent molecular weight according to brain region as well as by APOE and/or dementia status. We further found that antibodies to TMCC2 detect a pathological feature of Down syndrome AD that binds amyloid‐sensitive dyes, previously referred to as ‘fibrous plaques’ [5] or ‘bird's nest plaques’ [6], but without being previously assigned to a protein.

## 2 MATERIALS AND METHODS

### 2.1 Human brain

All procedures performed in studies involving human tissue were in accordance with the ethical standards of the 1964 Helsinki Declaration and its later amendments. Ethical approval was granted by the Brains for Dementia Research City and East National Research Ethics Service committee (08/H0704/128+5) and specimens provided by the Manchester Brain Bank, Salford, UK, and London Neurodegenerative Diseases Brain Bank—King's College London. Cases were anonymized, but information is provided regarding APOE genotype, sex, age at death and neuropathology (Table 1).

**TABLE 1:** Case characteristics.

Specimen characteristics								
Code	ID	Sex	Age	Clinical diagnosis	Pathological diagnosis 1	Pathological diagnosis 2	Braak stage	APOE genotype
A	BBN_3410	M	70	Dementia	AD	Very severe CAA	IV	33
B	BBN_3421	M	84	Cognitively normal	Moderate SVD		0 and I	33
C	BBN_3466	F	71	AD	Severe AD		VI	44
D	BBN_3472	M	73	AD	AD	Moderate SVD	VI	44
E	BBN_6080	F	81	AD	AD		IV and V	33
F	BBN_15257	M	77	FTD/PNFA	AD		IV and V	33
G	BBN_15309	M	77	Vascular dementia	AD		V	33
H	BBN_18819	F	89	Dementia	AD	Moderate SVD	V and VI	44
I	BBN_20005	M	85	Normal	Age changes only	Moderate SVD	0 and I	33
J	BBN_21395	M	69	AD	AD	Isocortical DLB	V and VI	44
K	BBN_24212	F	82	Control	Age changes only	Mild SVD	II	33
L	BBN_25109	F	79	Dementia/AD	AD		VI	44
M	BBN005.29168	M	90	Control	Normal for age	Mild SVD	I and II	33
N	BBN005.29398	M	84	Control	Normal ageing	Mild to moderate SVD	II	33
O	BBN005.30048	M	80	Vascular dementia	AD		V	33
P	BBN_9608	F	62	AD familial APP V717I			unkn	33
Q	BBN_13861	F	69	AD familial APP V717I	Dementia with Lewy bodies		unkn	34
R	BBN_13890	M	61	AD familial APP V717G	AD		unkn	34
S	BBN_13932	F	55	AD familial APP V717I	AD	Vascular malformation of the thalamus	unkn	unkn
T	BBN_13949	M	70	AD familial APP V717I	AD	Dementia with Lewy bodies	unkn	33
U	BBN_14050	F	59	AD familial APP V717I	AD	Dementia with Lewy bodies	unkn	33
V	BBN_169954	M	54	Down syndrome	AD		VI	unkn
W	BBN_16273	F	56	Down syndrome	AD	CAA	VI	unkn
X	BBN002.33767	F	54	Down syndrome	AD	CAA	unkn	unkn

Abbreviations: 33, homozygous for APOE3; 44, homozygous for APOE4; AD, Alzheimer's disease; CAA, cerebral amyloid angiopathy; DLB, dementia with Lewy bodies; FTD, frontotemporal dementia; PNFA, progressive non‐fluent aphasia; SVD, small vessel disease; unkn, unknown.

#### 2.1.1 Western blots and immunofluorescence

Homogenates were prepared from frozen brain tissue using an electrical homogeniser and sodium dodecylsulphate (SDS)‐containing buffer (4% SDS, 20% glycerol, 10% 2‐mercaptoethanol and 0.125 M Tris HCl, pH 6.8.) containing protease inhibitors (Complete, Boehringer Mannheim), followed by heating to 95°C for 5 min and centrifugation at 15,000*g* for 10 min. We specifically note that efficient recovery of TMCC2 required brain homogenates to be incubated with SDS prior to centrifugation; TMCC2 was not efficiently extracted from whole brain tissue by non‐ionic detergents such as Triton X‐100 or NP40. Rabbit anti‐TMCC2 antibodies 94 and 11193 were raised against recombinant TMCC2 as previously described [1] and used at 1/300 for tissue staining in 10% normal goat serum and 1/1000 for Western blots in 5% skim milk powder. sodium dodecylsulphate–polyacrylamide gel electrophoresis (SDS–PAGE) gels were 10% acrylamide and loaded with 1 mg of original tissue equivalent per lane. Data collection and quantification of band intensities for western blots was performed using the LI‐COR Odyssey platform: ratios of TMCC2 isoforms were estimated within lanes, and comparisons of total TMCC2 levels were made against a serially diluted pool consisting of equal proportions of all relevant samples loaded onto each gel; statistical calculations used Graphpad Prism. Anti‐Aβ antibody 4G8 was purchased from Chemicon (Temecula, CA, USA) and used at 1/300. Anti‐phospho‐tau antibody AT8 and anti‐tubulin antibody DM1A were purchased from ThermoFisher (Loughborough, UK) and used at 1/200 and 1/10,000, respectively; mouse monoclonal antibody C1/6.1 to the APP intracellular domain was purchased from Covance; and the mouse monoclonal antibody 22C11 to the APP ectodomain was purchased from Invitrogen.

For immunostaining, 7‐μm‐thick sections were cut from formalin‐fixed paraffin‐embedded blocks from the same cases as used in immunoblot protocols. Following dewaxing, antigen retrieval was performed with 80% formic acid for 20 min, rinsing in water, microwaving to boiling in sodium citrate pH 6.0 and kept in hot citrate buffer for 10 min following heating. Blocking was performed with normal goat serum (DAKO, Cambridgeshire, UK) for 1 h at room temperature. For dual immunostaining, anti‐TMCC2 was used at 1/300, and 4G8 or C1/6.1 at 1/500. Secondary antibodies were Alexa‐Fluor‐488‐conjugated goat anti‐rabbit and Alexa‐Fluor‐546 or Alexa‐Fluor‐568‐conjugated goat anti‐mouse, from Invitrogen, and incubated for 1 h at room temperature. Methoxy‐X04 was used at 10 μM for 20 min in phosphate‐buffered saline and autoflourescence quenched with Sudan black. Fluorescent images were collected on a Zeiss Axio Imager Z2 LSM 800 microscope and processed using Zeiss ZEN software and ImageJ.

#### 2.1.2 Quantification of co‐localization

Co‐localization of TMCC2 and APP in control and late onset AD tissue was performed using the Zeiss ZEN Analyzer software suite to estimate Mander's Overlap Coefficient with a background threshold set using the method of Costes. The data presented for each case represent the mean of an average of six 319.45 μm × 319.35 μm images each. For the post hoc quantification of dystrophies associated with dense‐cored plaques showing co‐immunostaining for TMCC2 and APP, to avoid bias, all previously captured images (5–12319.45 μm × 319.35 μm images for each case) were examined for the presence of dense‐cored plaques, which were then scored for the presence of adjacent dystrophies and whether such dystrophies displayed co‐staining for TMCC2 and APP. As early onset AD cases presented with a greater density of plaques than late onset AD cases, data are presented for each case on a per‐plaque rather than per‐area basis; statistical calculations were performed on a per‐case basis.

### 2.2 Statistical treatments

The current study represents, to our knowledge, the first examination of the TMCC2 protein in the human brain with AD. There were therefore no prior quantitative expectations and statistical tests were applied post hoc. For the statistical significance of differences in TMCC2 levels, isoforms by APOE genotype and brain region were estimated by one‐way analysis of variance with Tukey's multiple comparison test, or Student's *t*‐test. Calculations were performed using GraphPad Prism. Normality was determined using the Shapiro–Wilk test and shown to hold for all categories.

### 2.3 Accession numbers

TMCC2: Uniprot O75069, TMCC2_HUMAN; APP: Uniprot P05067, A4_HUMAN; ApoE: Uniprot P02649 and APOE_HUMAN.

## 3 RESULTS

### 3.1 TMCC2 immunoreactivity in the brain of non‐demented controls and its association with the pathology of late onset Alzheimer's disease

To investigate TMCC2 in late onset AD and whether this is influenced by APOE genotype, we examined TMCC2 protein levels and cellular distribution in five APOE3 homozygous cognitively healthy human brains with an average age of 85 (range 82–90), five AD cases homozygous for APOE3 pathologically diagnosed with AD (Braak stages IV and V) with an average age of 77 (range 70–81), and five AD cases homozygous for APOE4 and diagnosed with AD (Braak stages V and VI), with an average age of 76 (range 71–89). Case details are provided in Table 1.

**FIGURE 1:**
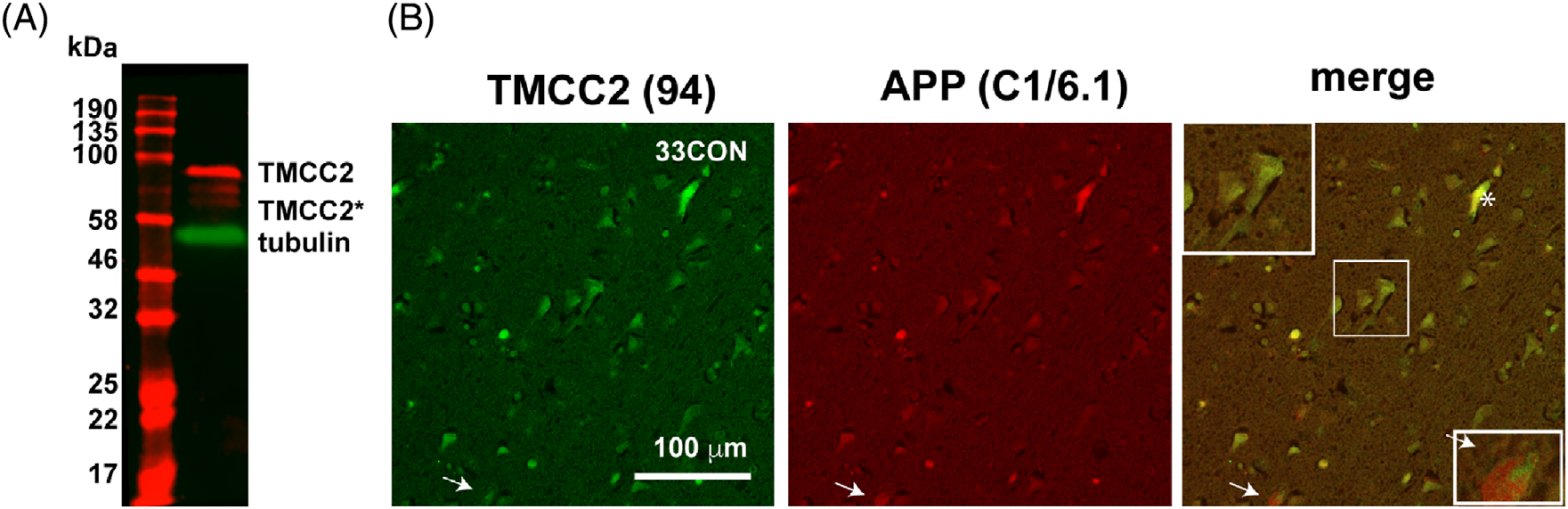
Expression of transmembrane and coiled‐coil 2 (TMCC2) in the human brain detected using antibody 94. (A) western blot of human superior temporal gyrus for TMCC2; (B) Table 1, case B. dual immunofluorescent detection of TMCC2 (green) and amyloid protein precursor (APP) (antibody C1/6.1, red) in a non‐demented APOE3 homozygous temporal gyrus revealing co‐localization of TMCC2 and APP. An enlarged image of neuronal soma is shown in the boxed inset. Arrow and inset in (B) indicates rare staining for APP that is not co‐incident with staining for TMCC2, the asterisk indicates a blood vessel staining for both TMCC2 and APP.

Using a rabbit antibody previously validated against mouse and rat brain‐derived TMCC2 (antibody 94) [1], we detected a set of three bands in human brain homogenates by western blot. All bands migrated with an apparent molecular weight close to or greater than that predicted from the amino acid composition of TMCC2 (77.5 kDa; Figure 1A). A similar pattern was observed using a separate independently generated antibody (antibody 11193), which, however, had reduced specificity compared to antibody 94 (Figure S1A).

Immunohistochemical staining for TMCC2 in the temporal cortex showed primarily a neuronal pattern (Figure 1B), consistent with previous detection in primary neurons [1] and proposed roles of TMCC2 in endomembrane systems [1, 7]. TMCC2 forms a complex with APP in transfected human cells and in the rat brain, where a large fraction of TMCC2 exists in a complex with APP [1]. We therefore examined human brain sections for both TMCC2 and APP by double immunofluorescence using antibody 94 to TMCC2 and antibody C1/6.1, which binds the C‐terminus of APP (residues 676–695 of APP_695_ [8]). We found a high degree of similarity between the staining patterns for both (Figure 1B). Similar results were obtained using antibody 22C11, which recognises the N‐terminal ectodomain of APP (Figure S1B). In non‐demented brains, TMCC2 immunoreactivity was rarely distinct from that for APP, whilst APP staining exists predominantly together with TMCC2 but is occasionally separated (Figure 1B, arrow), suggesting a dynamic association between TMCC2 and APP. Association of TMCC2 with APP may not be limited to the central nervous system: TMCC2 and APP immunoreactivity was also detected in the lumen of blood vessels (Figure 1B, asterisk), consistent with high expression of APP in platelets [9] with likely co‐expression of TMCC2 as TMCC2 may also function in haematopoiesis, being strongly linked to platelet and erythrocyte levels in genome‐wide association studies [10]; a blood‐specific isoform of TMCC2 (RefSeq NM_001297613) that may influence erythrocyte differentiation is translated from an alternate mRNA to yield a predicted 52 kDa protein [11] that would be detected by antibody 94. TMCC2 has been identified in several proteomic databases for platelets and mature erythrocytes [12, 13, 14].

**FIGURE 2:**
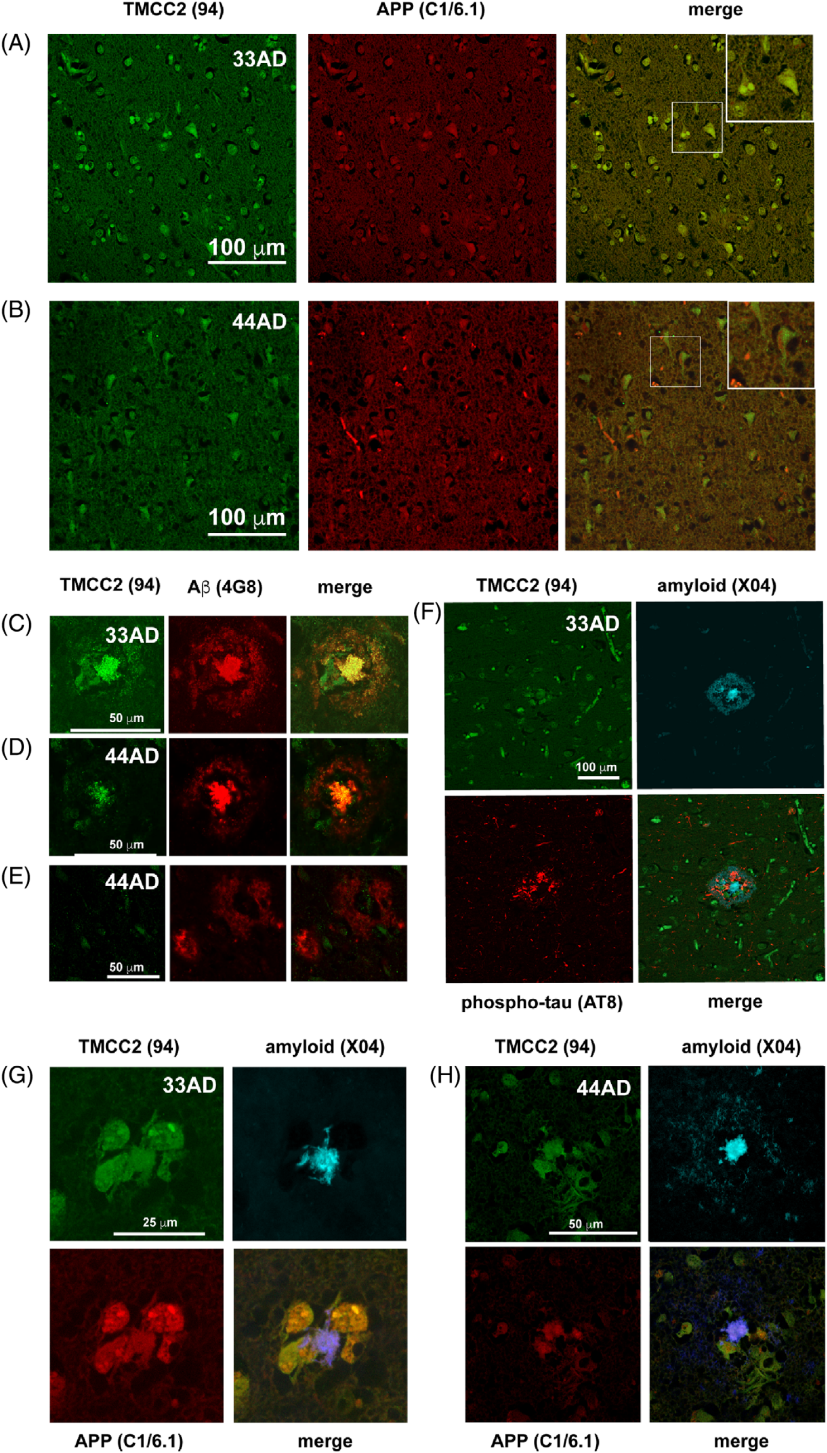
Association of transmembrane and coiled‐coil 2 (TMCC2) with late onset Alzheimer's disease (AD) pathology in human superior temporal gyrus. TMCC2 immunoreactivity was co‐incident with amyloid protein precursor (APP) immunoreactivity in the temporal gyrus of both APOE3 homozygotes (A, Table 1, case A) and APOE4 homozygotes (B, Table 1, case L) with AD. An enlarged image of neuronal soma is shown in the boxed insets of (A) and (B). TMCC2 was detected in dense‐cored amyloid plaques detected with antibody 4G8 in both APOE3 homozygotes and APOE4 homozygotes (C and D, Table 1, cases E and L, respectively), but not in diffuse amyloid plaques (E, Table 1, case L). TMCC2 was not detected in phospho‐tau‐positive neurites (F, Table 1, case E). TMCC2 and APP immunoreactivity co‐localised in APP‐positive dystrophic neurites surrounding dense‐cored plaques detected with methoxy‐X04 in both APOE3 homozygotes and APOE4 homozygotes with AD (G and H, Table 1 cases G and H, respectively).

In late onset AD brains from both APOE3 and APOE4 homozygotes, neurons had a shrunken appearance, with the distribution of TMCC2 immunoreactivity being similar to that seen in age‐matched non‐demented controls, though we subjectively noted a trend towards an increase of APP staining that was not co‐incident with that for TMCC2 (Figure 2A,B) that was reflected in a trend for a reduced co‐localization of APP and TMCC2 immunoreactivity, which however did not reach the conventional threshold for statistical significance, potentially caused by the small sample size (Figure S2C). To investigate whether TMCC2 associates with amyloid plaques, we stained AD cases using anti‐TMCC2 antibody 94 and anti‐Aβ (antibody 4G8). TMCC2 immunostaining was notable in the centre of dense core senile plaques from cases homozygous for either APOE allele but did not associate with the diffuse amyloid halo surrounding dense cores (Figure 2C,D and further figures below) or in diffuse plaques (Figure 2E and further figures below). Specificity of TMCC2 detection in dense‐cored plaques was confirmed using the separate TMCC2 antibody 11193 (Figure S2A).

We also examined if TMCC2 might associate with phospho‐tau‐filled neurites surrounding dense‐cored amyloid plaques detected using methoxy‐X04 (a derivative of Congo Red that labels amyloid) and the antibody AT8, which recognises tau phosphorylated at both serine 202 and threonine 205. We examined five late onset AD cases homozygous for APOE3, five late onset AD cases homozygous for APOE4 and five non‐demented cases homozygous for APOE3 (Table 1 cases A–O). We found no notable association of TMCC2 and AT8 immunoreactivity in any case (e.g., Figure 2F; a similar higher‐resolution image is shown in Figure S2D).

Senile plaques of AD exist in multiple morphologically and immunochemically distinct types with potentially variable origins. Among plaques having dense amyloid‐positive cores, a subset may contain phospholipid shells [15] and stain for cellular proteins [16, 17, 18], thus they may derive in part from degenerating neurons. We examined the distribution of TMCC2, APP and amyloid in late onset AD brain sections homozygous for either APOE3 or APOE4. This detected features strongly positive for both APP and TMCC2 and located adjacent to dense cores labelled with methoxy‐X04; these may represent axonal dystrophies (Figure 2G,H). The co‐localization of TMCC2 and APP immunoreactivity within these dense‐core‐associated APP‐positive dystrophies was almost ubiquitous; post hoc analysis showed that co‐localization of TMCC2 and APP immunoreactivity in these dystrophies was common in late onset AD across both APOE genotypes (c.f. Figures 2G and S2B) and more common in APOE3 than APOE4 cases, but rare in early onset AD (see below and Figure S4).

### 3.2 TMCC2 immunoreactivity in early onset Alzheimer's disease associated with mutation of APP residue Val717 and in Down syndrome

The above findings suggested an association of TMCC2 with late onset AD pathology. We extended our investigation to examine TMCC2 distribution in other examples of AD and examined early onset AD caused by mutation or over‐expression of APP. We examined five cases of early onset AD associated with the APP Val717 to Ile mutation (APP London), one case caused by the mutation of Val717 to Gly, as well as three cases of Down syndrome dementia where the gene for APP is present in excess.

**FIGURE 3:**
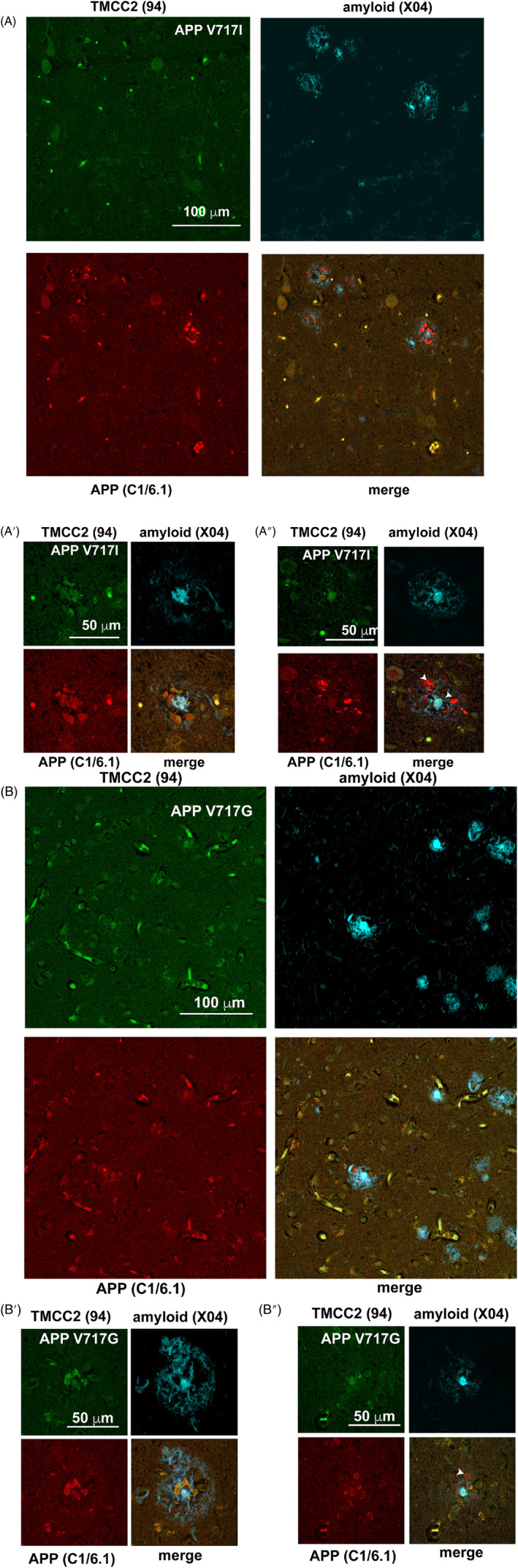
Transmembrane and coiled‐coil 2 (TMCC2) in early onset familial Alzheimer's disease (AD). TMCC2 associates with dense‐cored amyloid plaques and adjacent amyloid protein precursor (APP)‐positive dystrophic neurites in the temporal gyrus of familial AD associated with mutated APP. (A) APP V717I, Table 1, case V. (B), APP V717G, Table 1, case R. In cases with either APP mutant, abundant amyloid plaques were detected with methoxy‐X04 in the temporal gyrus (A and B). Dystrophic neurites surrounding dense‐cored plaques resembling those found in late onset AD and which stained for both TMCC2 and APP were found (A′ and B′), as well as plaques containing APP‐positive dystrophies that did not stain for TMCC2 (arrowheads in A″ and B″).

In early onset AD cases with APP‐Val717Ile, immunostaining of the temporal gyrus for TMCC2, APP and amyloid, showed extensive amyloid deposits and dense core plaques (Figure 3A). Dense core plaques where the surrounding dystrophic neurites stained for both APP and TMCC2 (Figure 3A′) were found, as were senile plaques surrounded by dystrophic neurites that stained for APP but not TMCC2 (Figure 3A″). Similar observations were made for the APP‐Val717Gly case (Figure 3B,B′,B″).

**FIGURE 4:**
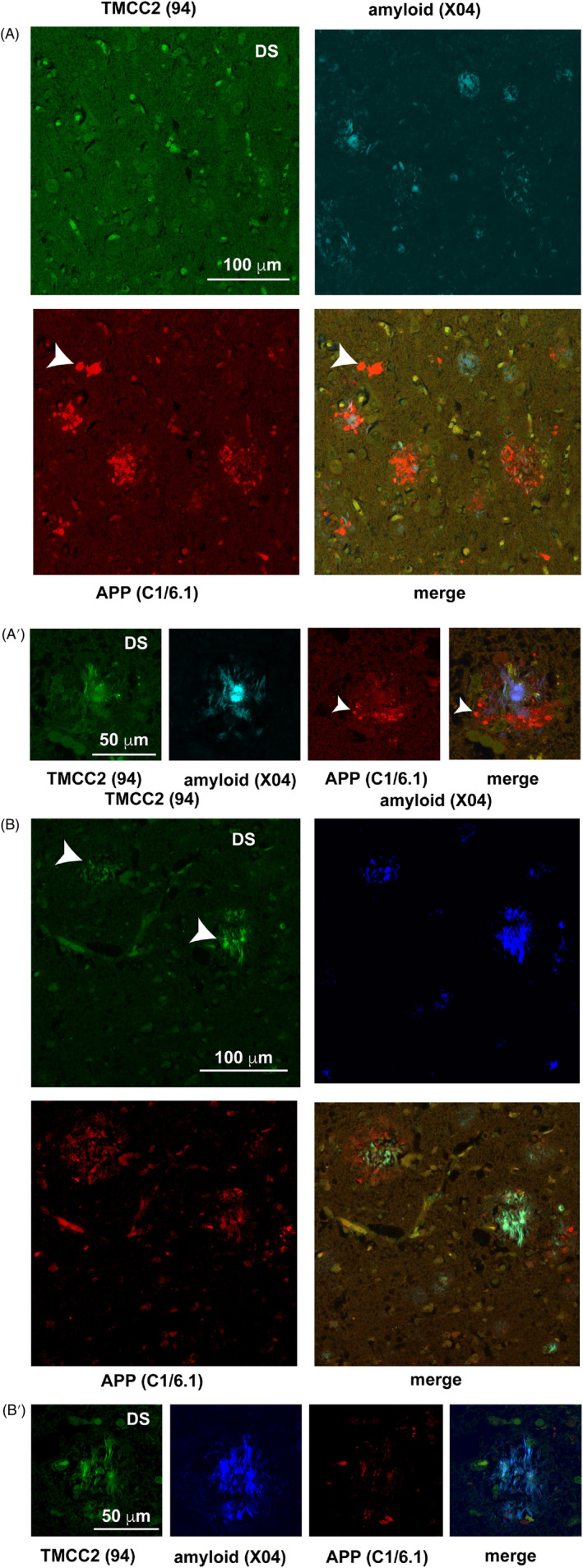
Transmembrane and coiled‐coil 2 (TMCC2) in Down syndrome (DS) and Alzheimer's disease (AD). (A) In Table 1, case V, abundant amyloid plaques and APP‐positive dystrophic neurites in Down syndrome temporal gyrus with a pathological diagnosis of AD. (A′) Table 1, case W, amyloid plaques resembling those found in late onset AD, that is, with a dense core detected with methoxy‐X04 and having adjacent dystrophic neurites staining for both APP and TMCC2 were found in all 3 cases. In all cases, frequent dystrophic neurites that stained for APP but not TMCC2 were seen (arrowheads in A and A′). In two cases (Table 1, X and W), lesions with putative amyloid as detected by methoxy‐X04 that were immunoreactive for TMCC2 was seen (arrowheads in B), see also a more highly magnified example in B′, both from Table 1, case W. An animation of B' is presented in Figure S5.

We investigated TMCC2 in three Down syndrome cases with a post‐mortem pathological diagnosis of AD (details in Table 1). In all cases, we detected abundant amyloid using methoxy‐X04, and dense‐cored plaques that were associated with dystrophic neurites that stained for both APP and TMCC2 in a manner similar to that found in familial and late onset AD (Figure 4A,A′). Thus, the presence of TMCC2 immunoreactivity in dense‐cored plaques of early onset AD cases examined here was comparable to that of the late onset AD cases shown above. However, co‐localization of TMCC2 and APP immunoreactivity seen in the putative axonal dystrophies bordering amyloid plaques of late onset AD (Figure 2G,H) was not observed in the early onset AD cases examined (Figure S4).

In two of the three Down syndrome cases examined (cases X and W; Table 1), we further found TMCC2 immunoreactivity to be associated with putative amyloid (as detected by methoxy‐X04) that was morphologically distinct from dense‐cored plaques and had a spicular or threadlike appearance (shown for case W in Figure 4B,B′). Figure 4B′ is a slice from a confocal stack; Figure S5 shows an animation of a 3D reconstruction of this stack that illustrates the interspersed nature of this pathological feature with cellular features and presumptive APP‐positive axonal dystrophies.

### 3.3 TMCC2 isoforms and their variation by brain region

**FIGURE 5:**
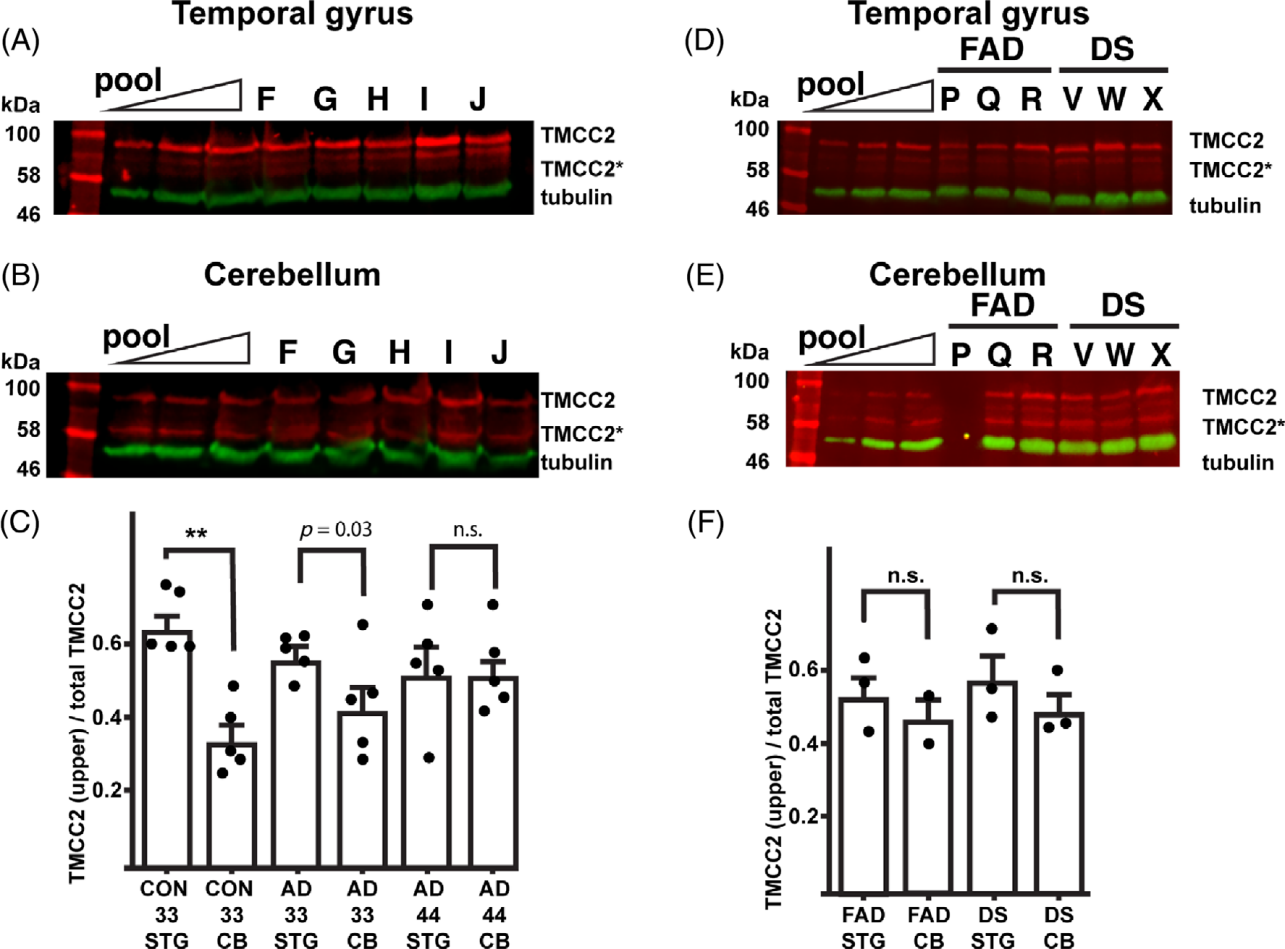
Transmembrane and coiled‐coil 2 (TMCC2) isoforms vary by brain region. Representative western blot of human superior temporal gyrus (A) or cerebellum (B) for higher migrating (TMCC2) and lower migrating (TMCC2*) TMCC2 in late onset Alzheimer's disease (AD) and matched controls detected using antibody 94; full blots are shown in Figure S3. (C) Quantification of the ratio of TMCC2 (uppermost band) to total TMCC2 in brain specimens stratified by brain region, APOE genotype and AD status; the statistical significance of differences was estimated by one way analysis of variance and Tukey's multiple comparison test (**, *p* < 0.005), or Student's *t*‐test (*p* = 0.03). (D, E) Western blot for TMCC2 in the temporal gyrus and cerebellum in early onset AD associated with a mutation of APP717 (FAD, familial AD) or Down syndrome (DS). (F) No significant differences (n.s.) in the ratios of TMCC2 (uppermost band) to total TMCC2 between the cerebellum (CB) and temporal gyrus (STG) were observed in these samples. Letters over lanes refer to cases described in Table 1.

Western blot for TMCC2 showed three bands that varied in relative intensity according to brain region (Figures 1 and 5), which potentially correspond to alternate TMCC2 isoforms, or alternate post‐translational modification of TMCC2. Within either the temporal gyrus or cerebellum, total levels of TMCC2 were unaffected by APOE or AD status in the cases assessed (Figure S3A,B). However, a larger fraction of cerebellum TMCC2 was found as the lower migrating band (TMCC2*) compared to TMCC2 from the superior temporal gyrus (Figure 5C). Densitometric quantification of the ratios of these putative isoforms within each of the 30 samples showed that 57.5 ± 2.7% of cerebellum TMCC2 was found as the lower migrating forms, compared to 42.5 ± 3% of temporal gyrus TMCC2 (*p* = 0.002 by two‐tailed Student's *t*‐test). This difference in ratios was observed in APOE3 homozygotes with or without AD, but not in APOE4 homozygotes with AD (Figure 5C; full blots are shown in Figure S3A,B). A trend for a lower ratio of higher apparent molecular weight TMCC2 to lower apparent molecular weight TMCC2 was observed between APOE3 homozygous controls versus APOE3 homozygous AD cases, but did not meet the commonly accepted threshold for statistical significance of *p* = 0.05 (Student's *t*‐test *p* = 0.07 and *p* = 0.13 for temporal gyrus and cerebellum, respectively).

Frozen tissue was available from a subset of early onset AD cases and analysed as above. This showed similar variation in gel band patterns of TMCC2 between the temporal lobe and cerebellum as seen in late onset AD cases and controls (Figure 5D,E; full blots are shown in Figure S3C,D). Down syndrome temporal gyrus showed an increase in the centrally migrating form of TMCC2 compared to late onset AD and familial AD; however, the differences in ratios of TMCC2 forms between temporal gyrus and cerebellum did not reach statistical significance (Figure 5F), potentially because of sample numbers being underpowered.

Thus, the form of TMCC2 present in the human brain was found to differ according to brain region as well as according to APOE and/or dementia status.

## 4 DISCUSSION

Support for a physiologically relevant interaction between TMCC2 and APP lies in its conservation between the human, rat and *Drosphila* orthologue pairs, indicating that this interaction has supported normal brain function through 600 million years of evolutionary pressure. Native gel analysis of rat brain preparations showed TMCC2 to exist in a protein complex with APP [1]; mutation of the *Drosophila* orthologue of TMCC2, Dementin, caused aberrant metabolism of the *Drosophila* APP‐like protein and neurodegeneration, and neurological defects caused by ectopic expression of human APP in *Drosophila* were rescued by co‐expression of wild‐type Dementin [2]. Human TMCC2 and APP co‐immunoprecipitate from SH‐SY5Y cell lysates, and here we show that in non‐demented post‐mortem human brains, TMCC2 and APP have similar distributions that may be disrupted in AD. The current study thus adds to the support for a mechanistic role for TMCC2 in human AD pathogenesis, both through its association with APP in healthy brains, and as a constituent of human AD pathology.

TMCC2 was initially identified as a molecule that may link APOE status to APP processing [1]. In this first study of TMCC2 in the human brain, we observed an association of TMCC2 immunoreactivity with that of APP, complementing previous observations of TMCC2‐APP complexes in the rat brain and human cells [1]. We also observed that this association is influenced by the APOE genotype within neuronal dystrophies of late onset AD. The evolutionarily conserved nature of this interaction indicates that the physiological functions of TMCC2 and APP are expected to overlap. TMCC family members are found within endomembrane systems [1, 7], though they may also be present at the cell surface in vivo [19], perhaps affecting the biology of APP in cell–cell interactions and synaptogenesis. Emerging data point to converging roles in brain development and neuronal synapse homeostasis [2, 20] that may be disrupted by mutation of TMCC2 or its orthologues as well as potentially by its interaction with apoE4. During the preparation of this manuscript, it was revealed that targeted alleles of mouse TMCC2 indicate a role for TMCC2 in cell survival, where TMCC2 deficiency leads to selective adult‐onset cell death in the nervous system, including auditory hair cells [21], suggesting a role for TMCC2 in the survival of neural cells.

Given the association of TMCC2 with both APP and apoE, and the established roles of these two proteins in AD pathogenesis, we investigated TMCC2 distribution in 19 AD cases having different genetic susceptibilities, either five cases each of APOE3 or APOE4 homozygotes in late onset AD as well as in five age‐matched controls, together with early onset AD associated with Down syndrome (three cases) or familial AD associated with APP mutated at Val717 (six cases).

We found a large degree of concordance between the staining patterns for APP and TMCC2 in both late onset AD brains and age‐matched healthy controls (Figures 1, 2 and S2C). Dense‐cored plaques with adjacent dystrophic neurites positive for TMCC2 and/or APP were found in all cases of both early and late onset AD (Figures 2G,H, 3A′,B′ and 4A′). Qualitative differences in staining between early and late onset AD cases were however noted: in both Down syndrome and familial AD, dystrophic neurites that stained intensely for APP in neuritic plaques were frequent (Figures 3 and 4), whilst similar features were rare in late onset AD (Figure S2B). Post hoc quantification of dense‐cored plaque‐associated dystrophies co‐immunostaining for TMCC2 and APP showed that co‐staining was significantly more common in the late onset AD than in the early onset AD cases examined and in cases homozygous for APOE3 versus APOE4 (Figure S4). Such APP‐positive dystrophies are a long‐standing finding in Down syndrome and early onset AD and also serve as a marker of axonal injury in non‐AD pathology [22, 23, 24, 25, 26, 27], and it has been previously noted that they are relatively uncommon in late onset AD [28, 29, 30]. Dissociation of APP staining from that of TMCC2 in early onset AD may relate to mutation of APP or reflect a more aggressive neuronal dysfunction and disease progression in early compared to late onset AD. All forms of AD share disrupted metabolism of APP and/or its metabolites; however, mutated or excess APP is the primary driver of disease in the cases of early onset AD examined, whereas in late onset AD APOE status is the principal genetic risk factor, suggesting potential differences in underlying pathomechanisms despite similarities in clinical presentation.

We found the strongest association of TMCC2 with AD pathology in Down syndrome, where, in addition to being detected in dense‐cored plaques as in late onset AD, TMCC2 immunoreactivity was also associated with putative amyloid having a spicular or thread‐like appearance (Figure 4B,B′). Such prominent TMCC2‐ and methoxy‐X04‐positive but APP‐negative features were not observed in the 10 late onset AD cases nor in the six familial AD cases examined. Methoxy‐X04 and thioflavin S have similar amyloid‐binding properties in the brain [31], and we note a resemblance between this TMCC2‐immunoreactive putative amyloid and the thioflavin S‐binding putative amyloid of ‘birds nest plaques’ that was not reactive with antibodies to Aβ or tau shown by Ichimata et al. [6]. Our observations also recapitulate the variation in presentation of the similar Aβ‐negative thioflavin S‐binding ‘fibrous plaques’ as shown by Schmidt et al. [5]. Further investigations into the potential for TMCC2 to adopt a β‐pleated sheet amyloid conformation in Down syndrome, or indeed also within dense core plaques of familial and late onset AD may therefore be warranted.

TMCC2 is located on chromosome 1q32.1 and so is not directly implicated in trisomy 21; putative alteration of TMCC2 neurobiology in Down syndrome may therefore relate to over‐expression of APP, its proteolytic products, or be associated with one or more of the 200–300 other genes present on chromosome 21, which will also be present in excess. In this respect, it has been noted in mouse models of trisomy 21 as well as in iPSC‐derived Down syndrome neurons, that chromosome 21 genes other than APP exacerbate AD pathogenesis [32, 33, 34].

TMCC2 binds apoE in an isoform‐specific manner in vitro, and apoE modifies the impact of TMCC2 on APP processing in cultured cells [1], apoE also modifies Aβ pathology in humans and mice [35, 36]. In non‐demented controls and age‐matched late onset AD cases stratified by APOE genotype, we found an association of APOE genotype and/or dementia with variation in the apparent molecular weight of TMCC2 as assessed by SDS–PAGE (Figure 5), all TMCC2 bands migrated at or above the expected position for the full‐length 709‐amino acid protein, and thus may reflect differential post‐translational modification of TMCC2, though alterations in mRNA splicing cannot be excluded. This suggests an influence of APOE on the biology of TMCC2 in the human brain and perhaps, by extension also on APP, though an influence of neurodegeneration per se on TMCC2 is also considered, as we were unable to obtain age‐matched non‐demented APOE4 homozygotes.

Limitations of this study include its cross‐sectional nature and the number of cases examined within each of the groups investigated; late onset AD cases homozygous for APOE4 were also at more advanced Braak stages (V and VI) compared to those homozygous for APOE3 (Braak stages IV and V), and our case numbers were underpowered to stratify findings by sex. Together, these limitations reduced the statistical power for a comparison of TMCC2‐immunoreactive pathology with genetic influences on AD pathogenesis. Further limitations include being unable to stratify our data according to post‐mortem delay and that, by necessity, studies using human post‐mortem tissue are observational in nature, which precludes definitive inferences as to the mechanism(s) by which TMCC2 might influence AD pathogenesis. Nevertheless, across 19 cases of AD having different genetic risk profiles, we found an association of TMCC2 immunoreactivity with the pathology of AD, suggesting novel directions for investigation into the mechanisms by which TMCC2 and apoE may contribute to AD pathogenesis.

## AUTHOR CONTRIBUTIONS

*Conceptualization*: PCRH and GT. *Data curation*: PCRH. *Formal analysis*: PCRH, GT, CT and AK. *Funding acquisition*: PCRH and GT. *Investigation*: PCRH. *Methodology*: PCRH and CT. *Project administration*: PCRH and GT. *Resources*: PCRH, GT, CT and AK. *Software*: not applicable. *Supervision*: not applicable. *Validation*: PCRH and CT. *Visualisation*: PCRH. *Writing—original draft*: PCRH. *Writing—review and editing*: PCRH, GT, CT and AK.

## FUNDING INFORMATION

This work was supported by an award from the Medical Research Council MC_PC_17164 (Paul C. R. Hopkins and Guy Tear) and supported by Alzheimer's Research UK and the Alzheimer's Society, through the Brains for Dementia Research Programme. Funders had no role in the study design, collection, analysis and interpretation of data, writing of the report, or the decision to submit the article for publication.

## CONFLICT OF INTEREST STATEMENT

The authors declare no competing interests.

## ETHICS STATEMENT

Ethical approval was granted by Brains for Dementia Research City and the East National Research Ethics Service committee.

## Supporting information


**Data S1.**Supporting Information.

**Data S2.** Supporting information.

## Data Availability

All data used are presented within the manuscript and Supporting Information, and reagents are available on request.
