# Supplementary material for: Transmembrane and coiled‐coil 2 associates with Alzheimer's disease pathology in the human brain
Source: Brain Pathol. 2024 Jul 31;35(1):e13290. doi: 10.1111/bpa.13290 (PMC11669416; doi:10.1111/bpa.13290)
Supplement: Supplementary file 1 — Data S1.Supporting Information. [file BPA-35-e13290-s002.docx]

**Supplementary Figures**

**Figure S1A.** Western blot of human temporal cortex or cerebellum pools for TMCC2 using anti-TMCC2 antibody 11193 confirming specificity of the TMCC2 and TMCC2* bands detected by antibody 94 shown in Fig. 1A and Fig 5. STG, superior temporal gyrus; CB cerebellum.


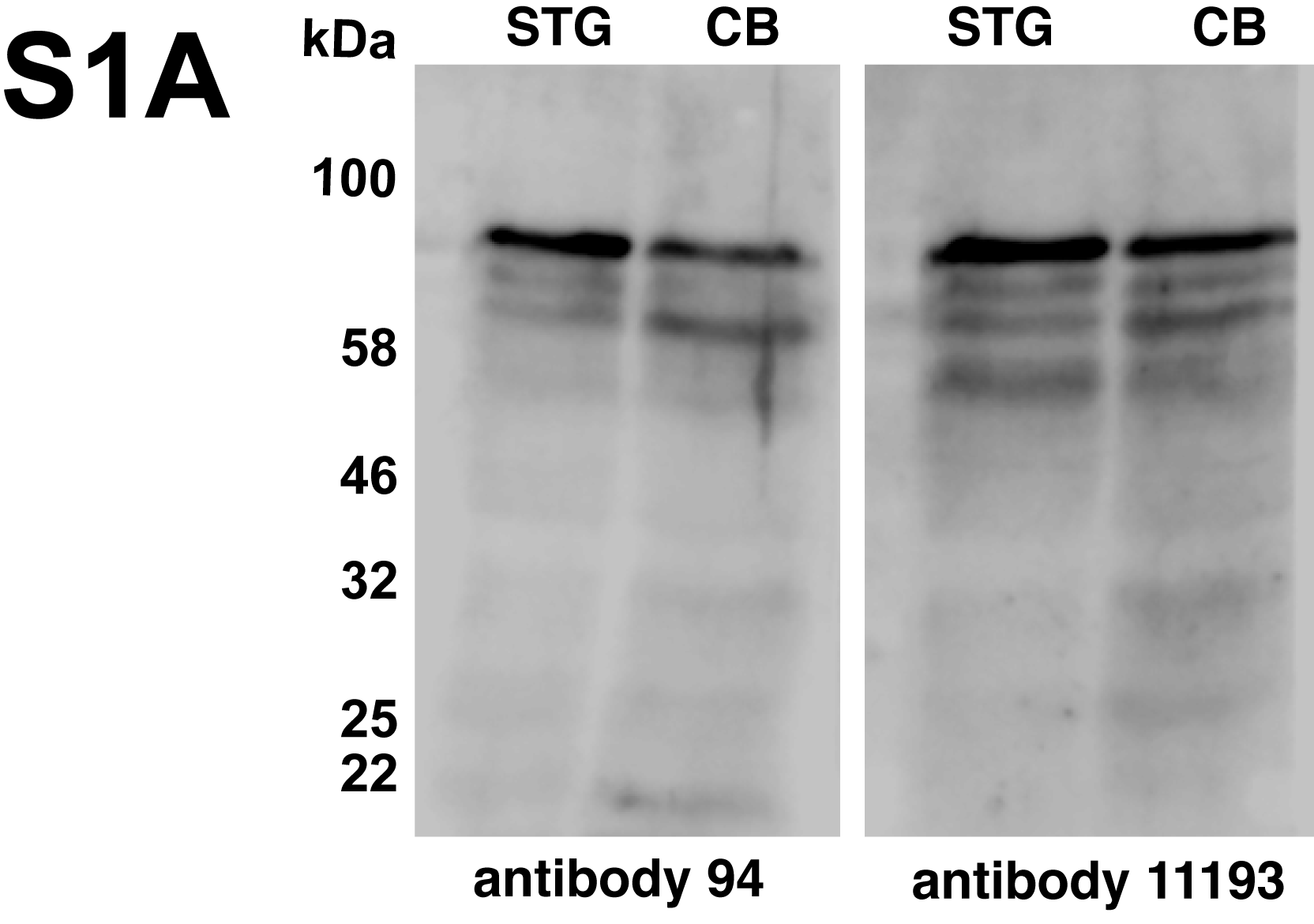


**Figures S1B to S1D.** Co-immunofluorescent detection of TMCC2 (antibody 94, green) and APP (antibody 22C11, red) in the temporal gyrus of a non-demented APOE3 homozygote (**S1B**), an APOE3 homozygote with AD (**S1C**) and an APOE4 homozygote with AD (**S1D**). In all amyloid was also detected with methoxy-X04 (blue).


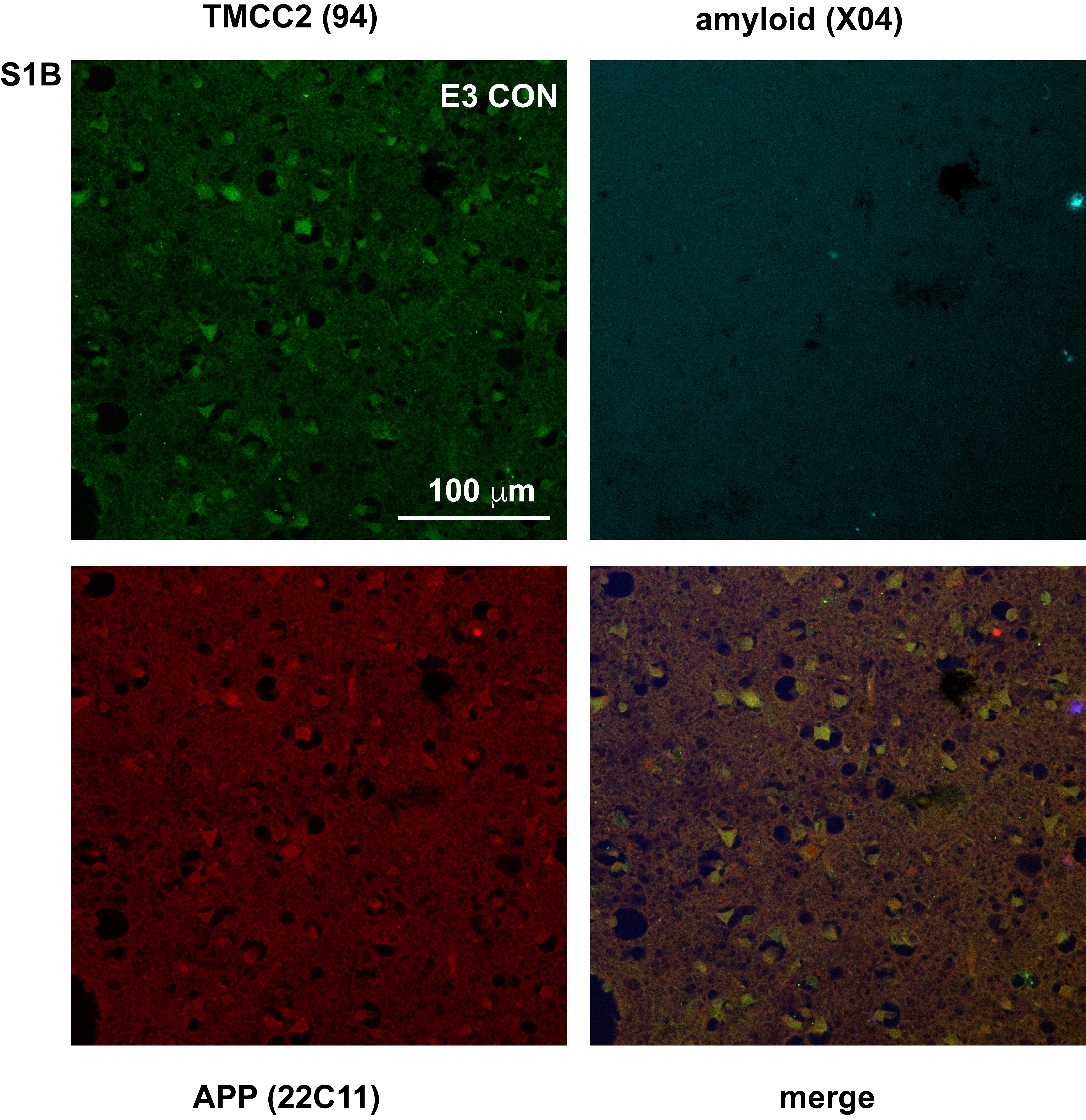


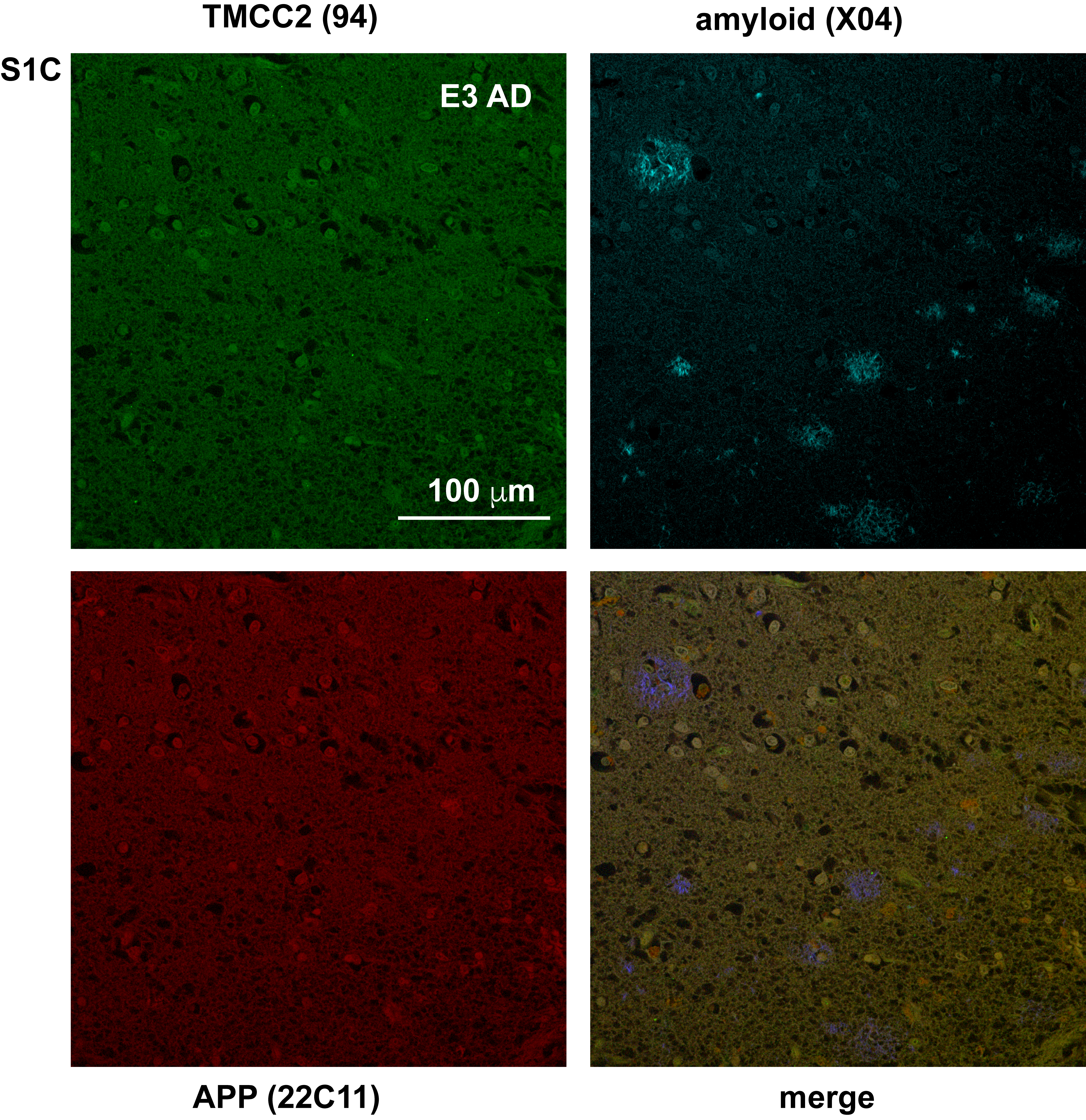


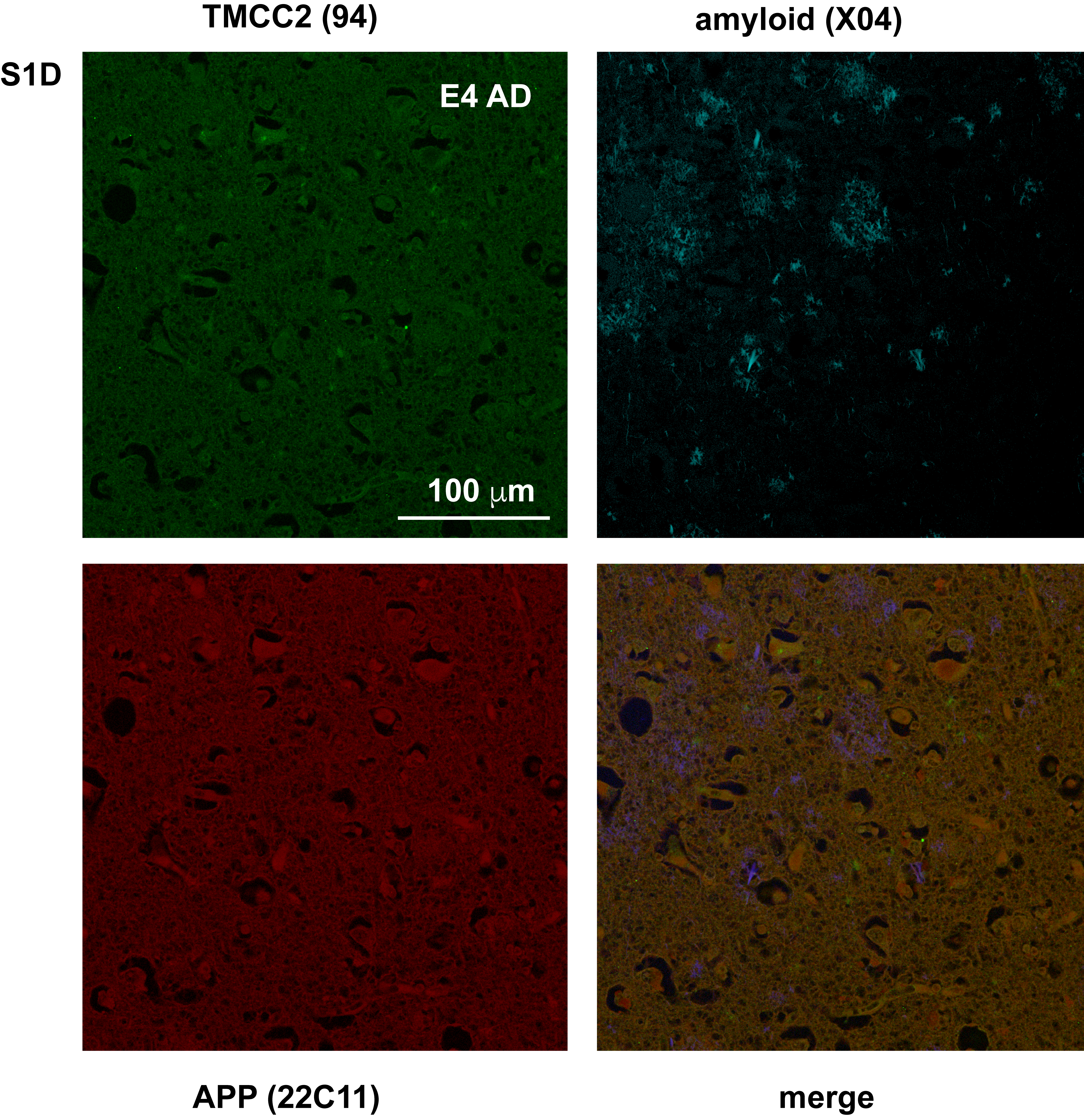


**Figure S2. S2A,** Table 1, case H, detection of TMCC2 using rabbit anti-TMCC2 antibody 11193 in dense-cored amyloid plaques identified using anti-Aβ antibody 4G8. **S2B**, Table 1, case L, example of rare separate staining for TMCC2 (antibody 94) and APP (antibody C1/6.1) in dystrophic neurites adjacent to dense cored amyloid plaques in temporal gyrus of late onset AD. **S2C**, quantification of co-localization TMCC2 and APP immunostaining in APOE3 homozygous controls (CON 33), and late onset AD homozygous for APOE3 (AD 33) or APOE4 (AD 44), bars represent the mean and standard error of the mean; differences in co-localization estimates were non-significant, one-way ANOVA P>0.05. **S2D**, co-immunostaining of a neuritic plaque in late onset AD (Table 1 case E) for phospho-tau (antibody AT8) and TMCC2 (antibody 94), with amyloid detected by methoxy-X04.

**
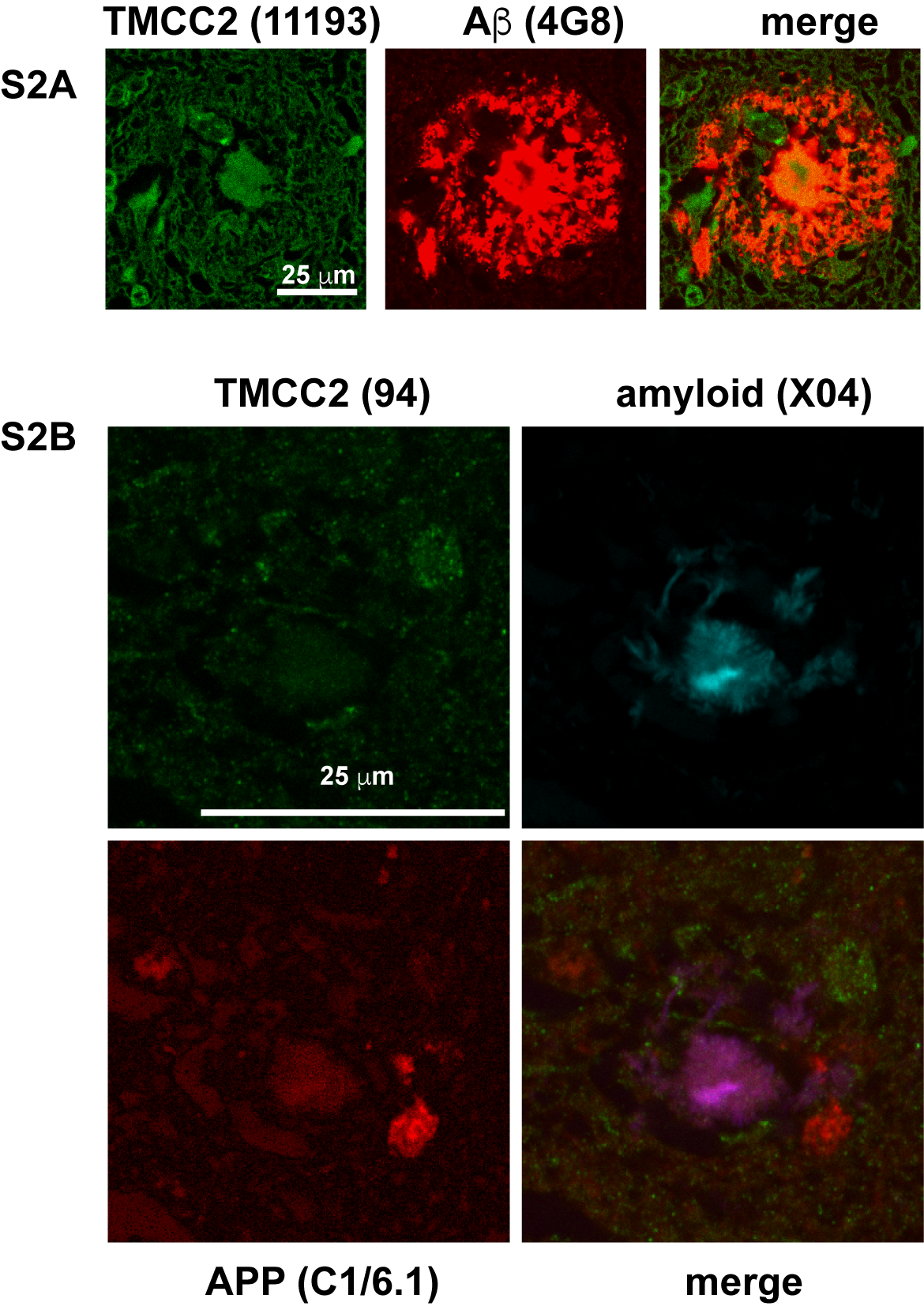
**


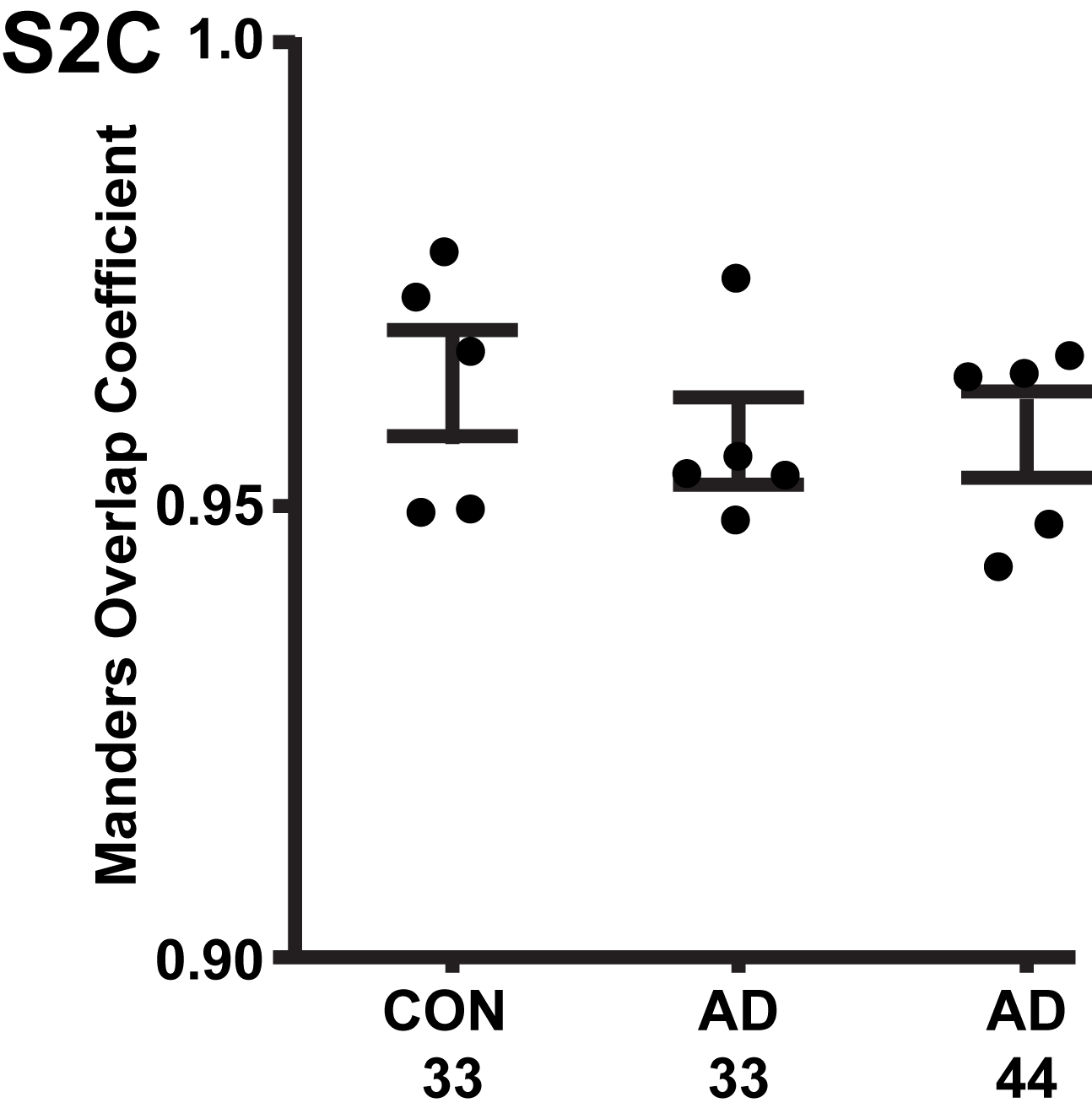


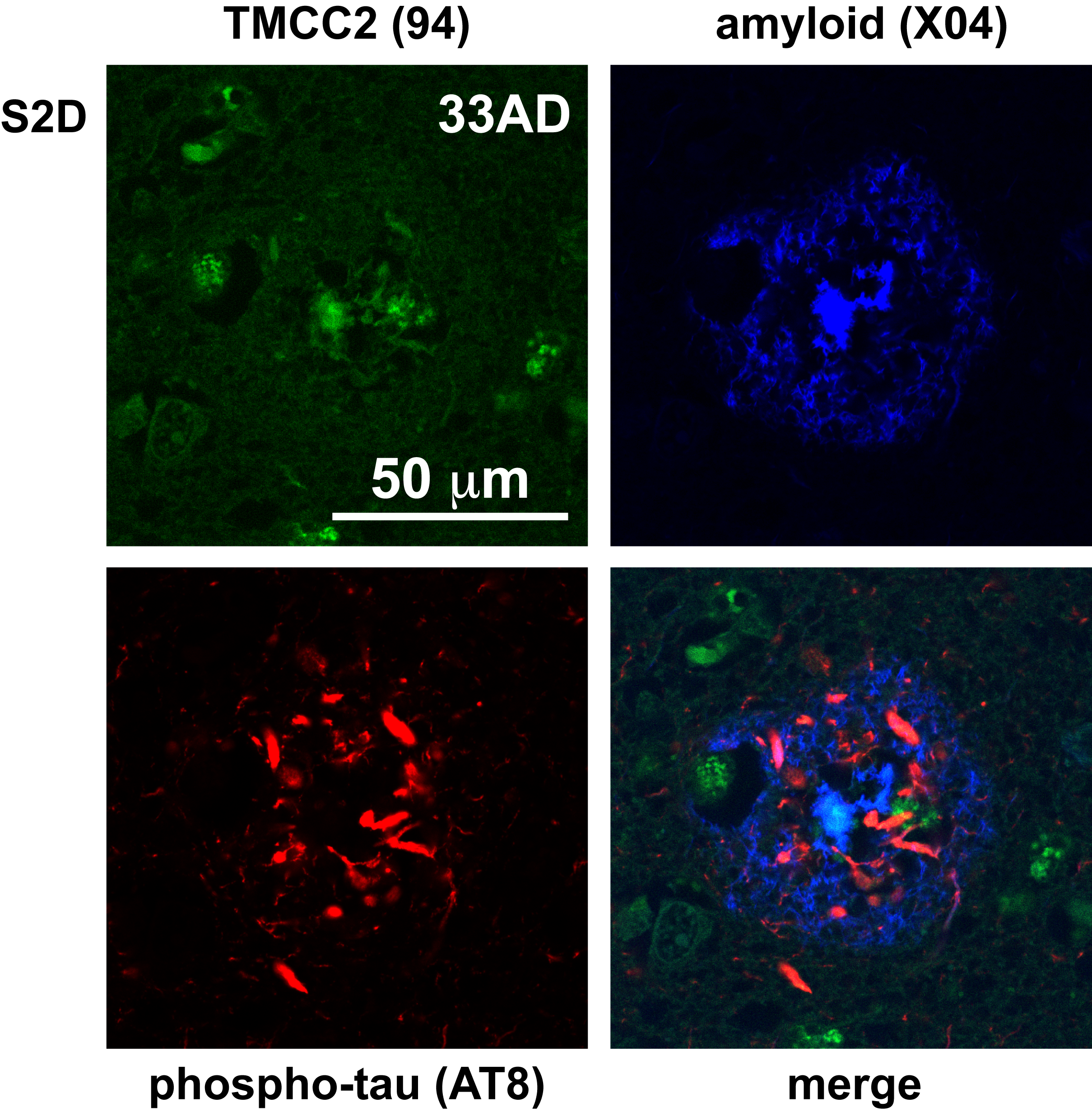


**Figure S3. Quantification of total TMCC2 levels according to brain region and AD status. S3A**, western blot and quantification of total TMCC2 levels from human temporal cortex using antibody 94. **S3B**, western blot and quantification of total TMCC2 levels from human cerebellum using antibody 94. Pools were created by combining equal quantities of all samples (temporal gyrus or cerebellum) and loaded at the equivalent of 0.5, 1 or 2 mg of original tissue. Bar charts show the relative levels of total TMCC2 for control APOE3 homozygotes, and AD cases homozygous for either APOE3 or APOE4 normalized between blots by reference to the band intensities of the pools; no significant association of APOE genotype or AD with total TMCC2 levels was found (p=0.63 for temporal gyrus and 0.24 for cerebellum, 1 way ANOVA). **S3C** and **S3D**, western blot of temporal gyrus and cerebellum, respectively, from early onset AD cases analysed as above. Frozen tissue from the cerebellum of case P was not available. Letters above lanes refer to cases described in Table 1.


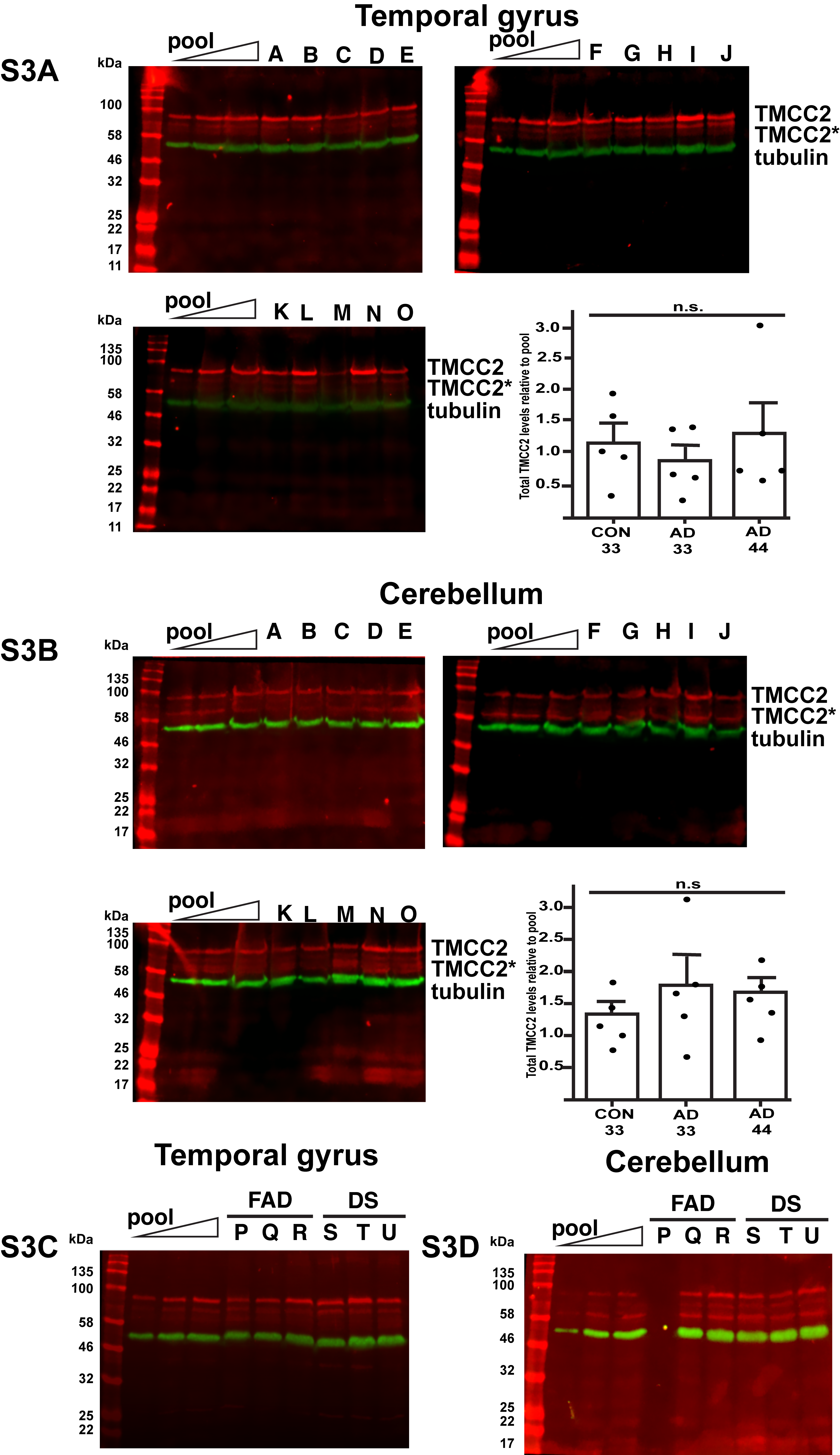


**Figure S4. Post-hoc quantification of dense-cored plaque-associated dystrophies showing co-localization of TMCC2 and APP**. Each point on the chart represents the proportion of dense-cored plaques showing co-localization of TMCC2 and APP in adjacent dystrophies as observed in 5 to 12 images for each case. The number of dense-cored plaques scored for APOE3 (AD 33) and APOE4 (AD 44) cases were 19 and 28, across 3 or 5 cases respectively. APOE3 AD cases F and G showed no dense cored plaques on post-hoc inspection. For Down syndrome (AD DS) and APP V717 (AD APP V717) cases, 50 and 211 dense cored plaques were scored, respectively. Statistical analysis was performed using 1-way ANOVA with Tukey’s Multiple Comparison Test, *** P = 0.0006; ** P < 0.005, * P < 0.05; independent comparison of APOE3 vs APOE4 dystrophies showing co-localization was significant by Student’s t-test (P = 0.03); other comparisons were non-significant (P > 0.05).


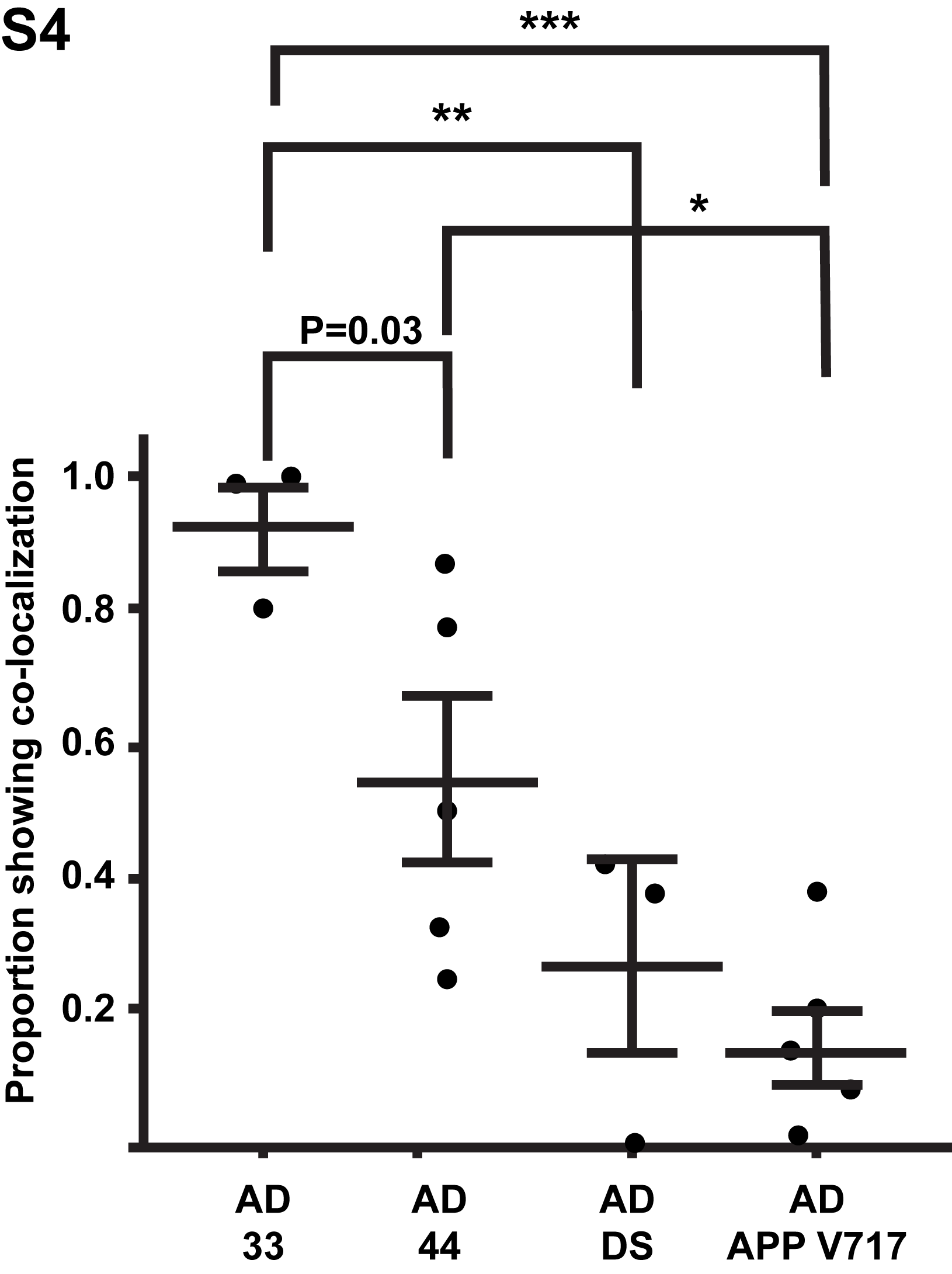


**Figure S5. Animation of a confocal stack associated with Figure 4B’.** Table 1, case W, 3D reconstructions of 1 μm optical sections of a TMCC2- and amyloid-positive plaque in a case of DS were animated using ImageJ.
