# Supplementary figures and images for: Transmembrane and coiled‐coil 2 associates with Alzheimer's disease pathology in the human brain
Source: Brain Pathol. 2024 Jul 31;35(1):e13290. doi: 10.1111/bpa.13290 (PMC11669416; doi:10.1111/bpa.13290)

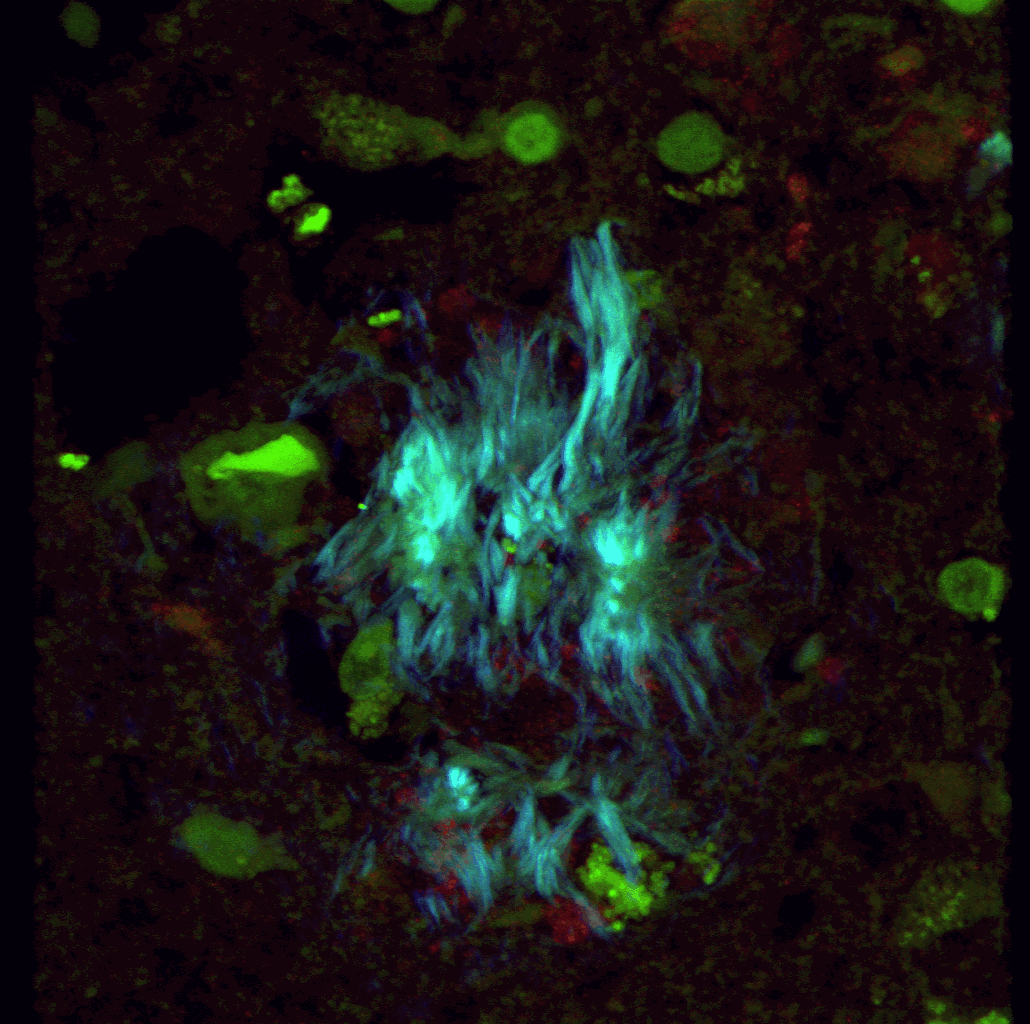

Supplement: Supplementary file 2 — Data S2. Supporting information. [file BPA-35-e13290-s001.gif]
